# Supplementary material for: Effective Clinical Pathway Improves Interprofessional Collaboration and Reduces Antibiotics Prophylaxis Use in Orthopedic Surgery in Hospitals in Indonesia
Source: Antibiotics (Basel). 2022 Mar 16;11(3):399. doi: 10.3390/antibiotics11030399 (PMC8944506; doi:10.3390/antibiotics11030399)
Supplement: Supplementary file 1 [file antibiotics-11-00399-s001.zip › antibiotics-1593892-Supplementary Material.pdf]

antibiotics

MDPI

Table S1. Indonesian version of CPAT questionnaire

Statement
Relationships among members
1. Anggota tim memiliki kepercayaan terhadap pekerjaan dan kontribusi setiap anggota dalam hubungannya dengan pelayanan pasien/klien.<br>Team members have confidence in the work and contribution of each member concerning patient/client care.
2. Anggota tim menghormati peran dan keahlian masing-masing.<br>Team members respect each other's roles and skills.
3. Tingkat saling menghormati dalam tim kami dapat meningkatkan kemampuan kami untuk bekerja sama.<br>The mutual respect level in our team can enhance our ability to work together.
4. Anggota tim peduli terhadap kesejahteraan satu sama lain.<br>Team members care about each other's well-being.
5. Bekerja dengan anggota tim lainnya merupakan hal yang menyenangkan.<br>Working with other team members is lively.
6. Kegiatan kebersamaan dapat meningkatkan efektivitas kerja tim.<br>Doing daily activities together can increase the effectiveness of teamwork.
7. Saling menghormati antara anggota tim meningkatkan kemampuan kami untuk bekerja sama.<br>Mutual respect between team members enhances our ability to work together.
8. Bekerja secara kolaboratif membuat sebagian besar anggota tim antusias dan tertarik terhadap pekerjaan mereka.<br>Working collaboratively makes most team members enthusiastic and interested in their work.
9. Pemimpin tim kami mendorong setiap anggota untuk berpraktik dalam seluruh lingkup profesional mereka.<br>Our team leaders encourage each member to practice within their entire professional scope.
Barriers in team collaboration
10. Kepemimpinan dalam tim menghambat para profesional untuk mengambil inisiatif dalam usaha mencapai tujuan pelayanan pasien/klien.<br>Team leadership hinders professionals from taking the initiative to achieve patient/client service goals.
11. Pemimpin tim kami tidak memperhatikan kekhawatiran dan persepsi anggota tim.

Our team leader unconcerns and inattentive the perceptions of team members.
12. Anggota tim merasa otonomi mereka terbatas dalam perawatan pasien/klien yang dapat mereka berikan.<br>Team members feel their autonomy is limited in the patient/client care they can provide.
13. Ketidaksetujuan antar anggota tim diabaikan atau dihindari.<br>Disagreements between team members are ignored or avoided.
14. Tim kami memiliki proses yang baku dalam manajemen konflik.<br>Our team has a standard procedure in conflict management.
Team relationships with the community
15. Tim kami telah membentuk kemitraan dengan organisasi masyarakat untuk mewujudkan luaran pasien/klien yang lebih baik.<br>Our team has formed partnerships with community organizations to achieve better patient/client outcomes.
16. Tim kami memiliki cara untuk mengoptimalkan koordinasi pelayanan pasien/klien dengan lembaga pelayanan masyarakat.<br>Our team has a way of optimizing the coordination of patient/client care with community service agencies.
17. Anggota tim kami berbagi informasi yang berhubungan dengan sumber daya komunitas.<br>Our team members share information related to community resources.
18. Janji temu pasien/klien dikoordinasikan sehingga mereka dapat bertemu beberapa pemberi layanan kesehatan dalam satu kunjungan.<br>Patient/client appointments are coordinated so they can meet multiple healthcare providers in one visit.
Team coordination and organization
19. Catatan kesehatan pasien/klien digunakan secara efektif oleh semua anggota tim sebagai alat komunikasi.<br>Patient/client health records are used effectively by all team members as a communication tool.
20. Saya percaya keakuratan informasi yang dilaporkan diantara anggota tim.<br>I trust the accuracy of the information reported among team members.
21. Ketika ada anggota tim yang tidak setuju, semua pandangan dipertimbangkan sebelum keputusan diambil.<br>When a team member disagrees, a decision is made by considering all member's views.
22. Informasi yang relevan berkaitan dengan perubahan status atau rencana perawatan pasien/klien dilaporkan kepada anggota tim yang sesuai dengan waktu yang tepat.<br>Relevant information related to patient/client status or treatment plans changes is reported to appropriate team members on time.

23. Kekhawatiran pasien/klien ditangani secara efektif melalui pertemuan rutin dan diskusi tim.<br>Patient/client concerns are handled effectively through regular meetings and team discussions.
24. Anggota tim memiliki tanggung jawab untuk berkomunikasi dan menyediakan keahlian mereka dengan cara yang asertif.<br>Team members have a responsibility to communicate and assertively provide their expertise.
25. Terdapat kejelasan mengenai siapa yang bertanggung jawab untuk aspek-aspek dalam rencana perawatan pasien/klien.<br>There is clarity on who is responsible for aspects of the patient/client care plan.
26. Informasi yang relevan dengan rencana pelayanan kesehatan pasien diberikan kepada pasien/klien.<br>Information relevant to the patient's health care plan is provided to the patient/client.
27. Tim kami sudah mengembangkan strategi komunikasi yang efektif untuk saling berbagi tujuan dan hasil tatalaksana pasien/klien.<br>Our team has developed effective communication strategies to share patient/client treatment goals and outcomes.
28. Setiap anggota tim bertanggung jawab terhadap keputusan dan hasil tim.<br>Each team member is responsible for team decisions and results.
29. Anggota tim merasa nyaman dalam memberikan advokasi terkait pasien/klien.<br>Team members feel comfortable advocating for patients/clients.
30. Pertemuan tim kami memberikan kesempatan yang terbuka, nyaman dan aman untuk membahas kekhawatiran.<br>Our team meetings provide an open, convenient and safe opportunity to discuss concerns.
31. Anggota tim bertanggung jawab terhadap pekerjaan mereka.<br>Team members are responsible for their work.
32. Anggota tim bertemu tatap muka dengan pasien yang dirawat oleh tim.<br>Team members meet face-to-face with patients treated by the team.
Decision making and conflict management
33. Dalam tim kami, penetapan keputusan akhir terkait pelayanan pasien/klien berada di tangan dokter.<br>In our team, the final decision regarding patient/client care is decided by the doctor.
34. Dalam tim kami, ada masalah yang secara teratur perlu dipecahkan oleh seseorang dengan posisi yang lebih tinggi.<br>Within our team, some problems regularly need to be solved by someone in a higher position.
Leadership

35. Pemimpin tim kami mencontohkan, menunjukkan dan mengadvokasi praktik baik yang berpusat pada pasien/klien.<br>Our team leaders exhibit and advocate for patient/client-centered good practice.
36. Kepemimpinan dalam tim menjamin bahwa peran dan tanggung jawab dalam pelayanan pasien/klien didefinisikan dengan jelas.<br>Team leadership ensures that roles and responsibilities in inpatient/client care are clearly defined.
37. Kepemimpinan dalam tim mendukung adanya peluang pengembangan interprofesional.<br>Team leadership supports interprofessional development opportunities.
38. Tim kami memiliki proses penilaian antara sejawat.<br>Our team has a peer-to-peer assessment process.
39. Langkah-langkah tersedia untuk mengidentifikasi dan merespon masalah secara cepat.<br>There are some steps to identify and respond to problems fast.
Mission, goals and objectives
40. Misi tim kami mewujudkan pendekatan kolaboratif interprofesional dalam pelayanan pasien/klien.<br>Our team's mission is to embody an interprofessional collaborative approach to patient/client care.
41. Tujuan tim kami jelas, bermanfaat dan sejalan dengan praktik saya.<br>Our team goals are clear, beneficial, and related to my practice.
42. Tujuan utama tim kami adalah membantu pasien/klien dalam mencapai tujuan pengobatan.<br>Our team's foremost goal is to help patients/clients achieve their treatment goals.
43. Rencana pelayanan dan tujuan pengobatan pasien/klien memasukan paduan praktik baik dari berbagai profesi.<br>Service plans and patient/client treatment goals incorporate the integration of good practices from various professions.
44. Misi dan tujuan tim kami didukung oleh sumber daya yang cukup (keterampilan, dana, waktu, ruangan).<br>Our team's mission and goals are supported by sufficient resources (skills, funds, time, space).
45. Semua anggota tim memiliki komitmen terhadap praktik kolaboratif.<br>All team members are committed to collaborative practice.
46. Anggota tim kami memiliki pemahaman yang baik mengenai rencana pelayanan dan tujuan pengobatan pasien/klien.<br>Our team members have a good understanding of the service plan and patient/client treatment goals.
47. Ada keinginan nyata diantara anggota tim untuk bekerja secara kolaboratif.

There is a real solicit among team members to work collaboratively.
48. Kepemimpinan dalam tim memastikan bahwa semua profesi yang dibutuhkan memiliki peran dalam tim.<br>Team leadership ensures that all required professions have a role in the team.
Patient involvement, responsibility and autonomy
49. Jika pasien meminta, maka keluarga dan dukungan lain dimasukkan dalam rencana pelayanan.<br>If the patient requests, then family and other support are included in the service plan.
50. Pasien/klien dianggap sebagai bagian dari tim pelayanan kesehatan.<br>Patients/clients are considered part of the health care team.
51. Anggota tim mendorong partisipan aktif dari pasien/klien dalam membuat keputusan pelayanan.<br>Team members encourage active patient/client participation in the decision-making process.
52. Dokter mengambil peran sebagai penanggung jawab utama untuk keputusan dan luaran dari tim.<br>Doctors have the role of primary responsibility for the decision-making and outcomes fulfillment of the team.
53. Para anggota tim mengakui aspek pelayanan dimana anggota profesi saya memiliki lebih banyak keterampilan dan keahlian.<br>Team members recognize aspects of service where members of my profession have more skills and expertise.

Likert scale categories: Strongly agree: 5, Agree: 4, Neutral: 3, Disagree: 2, Strongly disagree: 1
